# Hot Water Immersion as a Means to Prevent Cardiovascular Disease and Associated Mortality

**DOI:** 10.2174/011573403X319557240822094347

**Published:** 2024-08-23

**Authors:** Metodija Kjertakov, Aaron Petersen

**Affiliations:** 1 Institute for Health and Sport, Victoria University, Melbourne, Australia

**Keywords:** Cardiovascular disease, cardiovascular mortality, prevention, physical activity, passive heat exposure, hot water immersion

## Abstract

Physical activity is widely promoted as a preventive strategy against cardiovascular disease and death from this disease. However, the fact that some individuals are unable or unwilling to engage in physical activity highlights the need for alternative strategies. Passive heat exposure using hot water immersion could serve as an alternative to physical exercise, as it provides similar, if not greater, cardioprotection than physical activity. This perspective article presents evidence supporting our concept and provides recommendations for hot water immersion sessions.

## 1 INTRODUCTION

Based largely on the evidence of a strong and independent relationship between increased levels of physical activity and reduced risk of developing cardiovascular disease (CVD) and associated mortality [1-6], physical activity has been widely promoted as a preventive strategy against CVD and death from this disease [7-11]. The minimum dose of weekly physical activity recommended since 2008 is either 150 minutes of moderate-intensity aerobic physical activity or 75 minutes of vigorous-intensity aerobic physical activity [10], and it was recently reported that meeting these recommendations reduces the risk of CVD mortality by 35% [12]. While the cardiovascular benefits associated with physical activity are undeniable, there are individuals with physical disabilities and medical conditions who are deprived of those benefits due to the inability to engage in or perform physical activities at an intensity high enough to induce cardioprotective effects. There are also individuals who are unwilling or feel unsafe (*e.g*., older people) to get involved in physical activities [13].

One intervention that could serve as an effective alternative to physical activity in such individuals, as presented in this paper and suggested previously by other researchers [14-16], is passive heat exposure. Effective implementation of such an intervention requires an increase in body core temperature by at least ~1°C [15], which is easily achieved by either hot water immersion [17] or sauna bathing [18]. Furthermore, both heating methods have recently been associated with cardioprotection [19, 20]. However, the practical advantage of hot water immersion over sauna bathing is that it requires only a bathtub and hot water, and, as such, it can be conducted by many potential users in their homes [21]. Hot water immersion could also be suitable for use at aged care homes where health professionals can supervise its application to older people. Therefore, the current paper intends to promote passive heat exposure *via* hot water immersion as an effective alternative to physical activity in preventing CVD and death from this disease. First, we present evidence supporting our concept. Then, we provide recommendations for hot water immersion sessions. In some instances, we refer to sauna bathing studies since their findings are highly relevant to the current idea.

## 2 EVIDENCE THAT HOT WATER IMMERSION COULD ASSIST IN COMBATING CVD

Preventing the onset of CVD is considered the best treatment for this disease [22], and evidence that hot water immersion could serve that purpose emerged recently from an epidemiological study conducted in Japan by Ukai *et al.* [19]. At baseline, 30.076 apparently healthy men and women aged 40 to 59 completed a questionnaire that collected information about their demographic characteristics, health status, and tub bathing habits, and subsequently, were followed up for 19 years [19]. Based on the frequency of bathing, participants were first divided into three groups: two or fewer times a week, three to four times a week, or almost daily or daily. Then, they were also divided into groups bathing either in lukewarm, warm, or hot water. Although no information was provided about the duration of bathing sessions or water temperature in baths, it is well-known that typical Japanese-style hot bathing consists of sitting in a 40-42°C bath up to the shoulders for ~10 minutes [23]. Furthermore, a session like that is reported to increase body temperature by about 1°C [17].

The results of the study revealed that the hazard of developing CVD in individuals bathing in a hot tub almost daily or daily was 35% (95% CI: 0.44, 0.95) lower compared to those bathing in a hot tub two or fewer times per week, independently of the well-established CVD risk factors as well as several other potential confounders [19]. Noteworthy, the risk reduction of CVD associated with regular hot water bathing falls within the range of risk reduction (25% - 52%) reported with physical activity [2-4], supporting our idea that hot water immersion can be an effective alternative to physical activity in preventing CVD. Interestingly, regular warm water bathing also provided protection against CVD, albeit to a lesser extent than the respective hot water bathing group. The group bathing in warm water almost daily or daily exhibited a hazard of developing CVD that was 26% (95% CI: 0.61, 0.90) lower than the group bathing in warm water two or fewer times per week. Unfortunately, the lack of information on the characteristics of bathing sessions (*i.e.*, their durations and water temperatures) prevents further interpretation of these results. Furthermore, when Ukai *et al.* [19] examined the associations between the tub bathing frequency of all three groups combined and the risk of hypertension, diabetes, and hypercholesterolemia, they found that frequent bathing was associated with a 20% less chance of developing hypertension compared to bathing two or fewer times per week. Given that hypertension is considered the leading risk factor for CVD [24], the latter finding helps in part explain the risk-reduction effect of regular hot water bathing on CVD. However, due to the statistical approach used, the actual impact hot tub bathing has on the risk of hypertension is unclear. The ability of hot water immersion to decrease blood pressure is well-documented in randomised controlled trials [25-28], with the benefits being attributed to improved arterial compliance. Randomised controlled trials [27, 29] also showed beneficial effects of hot water immersion on other CVD risk factors, such as fasting blood glucose and cholesterol levels. Still, Ukai *et al.* [19] observed no association between regular bathing and the risk of diabetes or hypercholesterolemia. Again, for the same reason mentioned above, whether hot tub bathing impacted the risk of those two conditions remains unclear. Theoretically, using a hot water immersion protocol that, in addition to a beneficial effect on blood pressure, may minimise the risks of diabetes and hypercholesterolemia would have the most significant risk reduction potential of CVD. In that context, the minimum 'dose' of heat stress demonstrated to decrease fasting glucose, total cholesterol, and low-density lipoprotein cholesterol in chronic studies was 30 minutes of daily passive heat exposure that increased body temperature by ~1°C [30, 31]. In contrast, blood pressure appears to respond well to only 10 minutes of immersion in 40°C water [28].

Clearly, the results of Ukai *et al.* [19] have important public health implications. However, the limitation of their study is that they did not attempt to assess the potential benefits of hot water bathing on the risk of dying from CVD. The researchers do report a trend (*p* = 0.06) of reduced risk of sudden cardiac death (the only fatal CVD outcome studied) in individuals bathing almost daily or daily compared to those bathing two or fewer times per week [19], but we assume that the small sample size (*n* = 35) of this data set prevented them running further analyses. Elsewhere, in an epidemiological study conducted in Finland that followed 1688 men and women for 14 years, Laukkanen *et al.* [20] described the relationship between Finnish sauna bathing and CVD mortality. After adjustments for potential confounding factors, the hazard of CVD mortality in individuals who had four to seven sauna bathing (13 minutes at 74°C) sessions per week was 77% (95% CI: 0.08, 0.65) lower compared to those bathing in sauna (77°C) for only 10 minutes once a week. By comparison, as mentioned in the introduction, people who complied with 2008 physical activity guidelines experienced a reduced risk of CVD mortality by 35% [12]. Although it is unknown to what extent the findings of Laukkanen *et al.* [20] could be extended to hot water immersion, it is reasonable to expect that the latter method would considerably reduce the risk of dying from CVD. That notion is supported by the evidence that hot water immersion can induce the same physiological changes as those implicated in the cardioprotective effects of sauna bathing [20], including decreased blood pressure [25-28], improved vascular function [26], and reduced inflammation [27, 32]. In addition, hot water immersion could modify the risk of dying from CVD by increasing cardiorespiratory fitness (CRF). Indeed, the role CRF plays in cardioprotection is well-established [33], and the study by Bailey *et al.* [34] showed that immersion in 42°C water to the sternum for 30 minutes, thrice weekly, for only two months increased peak oxygen uptake (*i.e.,* the 'gold standard' measure of CRF) by 5% (~2 ml∙kg^-1^∙min^-1^) in healthy individuals. As an illustration, an increase in peak oxygen uptake by 2 ml∙kg^-1^∙min^-1^ decreases the risk of dying from CVD by 30% in healthy individuals [35] and 18% in patients with documented CVD [36]. Collectively, the evidence presented in this section (1) indicates that hot water immersion can minimise the risk of developing CVD to an extent similar to that associated with physical activity, and (2) suggests that hot water immersion has the potential to minimise the risk of dying from CVD. Still, the actual impact of hot water immersion on the risk of dying from CVD needs to be determined.

## 3 RECOMMENDATIONS FOR HOT WATER IMMERSION SESSIONS

First, the available evidence indicates that hot water immersion is safe for use in a range of populations, including older people [25, 37], individuals with conditions that impair thermoregulatory capacity [30, 38], and even patients with chronic congestive heart failure [17]. Nevertheless, users need to be made aware that hot water bathing, particularly Japanese-style bathing, is not risk-free. Indeed, staying submerged up to the shoulders for too long in 40-42°C water may drive body core temperature to the point of heat stroke [39-41]. Therefore, we recommend using the hot water immersion protocol described by Bailey *et al.* [34] in the preceding section rather than Japanese-style hot tub bathing. The magnitude of increase in core temperature (~1°C) induced by sitting for half an hour in a bath filled with 42°C water up to the sternum with arms outside the water is considerably below that (~4°C) shown to produce heat stroke [42, 43] but still high enough to promote the desired cardiometabolic and cardioprotective benefits. Using this hot water immersion protocol five days per week for at least a month should lead to measurable change in the earlier-mentioned health markers [28, 30, 31]. Additionally, its lifetime use is expected to minimise the risk of developing CVD or dying from it.

If staying submerged in water at 42°C for 30 minutes [34] is thermally challenging, taking ~2-minute breaks outside the bathtub every 10 minutes during immersion would relieve thermal discomfort and allow completion of the 30-minute hot water immersion session [44]. Alternatively, the water temperature in the bathtub could be lower (40°C), but this needs to be compensated by longer immersion time (1 hour) for users to experience an increase in core temperature like that associated with the former hot water immersion protocol [45]. Since hot water immersion may cause dizziness and syncope [46], users should not hurry to stand after finishing the bathing session. They should sit for a few minutes after getting out of the bath to allow blood pressure to normalise. Finally, hot water immersion may be contraindicated in patients with epilepsy, unstable angina pectoris, recent myocardial infarction, ischemic or decompensated heart failure, severe aortic stenosis, severe orthostatic hypotension, acute infectious or inflammatory conditions, and fever [47, 48].

## CONCLUSION

The evidence presented in this paper suggests that passive heat exposure *via* hot water immersion could be offered as a substitute for physical activity in preventing CVD and mortality from this disease to individuals who, for whatever reason, cannot adhere to the current physical activity guidelines.

## LIST OF ABBREVIATIONS

CRF: Cardiorespiratory Fitness
CVD: Cardiovascular Disease
